# Association between minimally invasive surgery and late seizures in patients with intracerebral hemorrhage: A propensity score matching study

**DOI:** 10.3389/fsurg.2022.949804

**Published:** 2022-10-13

**Authors:** Jiahe Lin, Ru Lin, Xianxian Li, Jiahe Ye, Yuchen Wang, Beining Zhang, Xinling Chen, Xinshi Wang, Shanshan Huang, Suiqiang Zhu

**Affiliations:** ^1^ Department of Neurology, Tongji Hospital, Tongji Medical College, Huazhong University of Science and Technology, Wuhan, China; ^2^ Department of Radiology, The First Affiliated Hospital of Wenzhou Medical University, Wenzhou, China; ^3^ The First Clinical Medical College, Wenzhou Medical University, Wenzhou, China; ^4^ Department of Neurology, The First Affiliated Hospital of Wenzhou Medical University, Wenzhou, China

**Keywords:** intracerebral hemorrhage, minimally invasive surgery, seizures, propensity score matching, risk factor

## Abstract

**Purpose:**

The association between minimally invasive surgery (MIS) for hematoma evacuation and late seizures after intracerebral hemorrhage (ICH) remains uncertain. We aimed to investigate whether MIS increases the risk of late seizures after ICH and identify the risk factors for late seizures in this patient subgroup.

**Methods:**

We retrospectively included consecutive inpatients diagnosed with ICH at two tertiary hospitals in China. The subjects were divided into the MIS group (ICH patients who received MIS including hematoma aspiration and thrombolysis) and conservative treatment group (ICH patients who received conservative medication). Propensity score matching was performed to balance possible risk factors for late seizures between the MIS and conservative treatment groups. Before and after matching, between-group comparisons of the incidence of late seizures were performed between the MIS and conservative treatment groups. Univariate and multivariate logistic regression analyses were used to identify independent risk factors for late seizures in MIS-treated patients.

**Results:**

A total of 241 and 1,689 patients were eligible for the MIS and conservative treatment groups, respectively. After matching, 161 ICH patients from the MIS group were successfully matched with 161 ICH patients from the conservative treatment group (1:1). Significant differences (*p* < 0.001) were found between the MIS group (31/241, 12.9%) and conservative treatment group (69/1689, 4.1%) in the incidence of late seizures before matching. However, after matching, no significant differences (*p* = 0.854) were found between the MIS group (17/161, 10.6%) and conservative treatment group (16/161, 9.9%). Multivariate logistic regression analysis revealed that cortical involvement (OR = 2.547; 95% CI = 1.137–5.705; *p* value = 0.023) and higher National Institutes of Health Stroke Scale (NIHSS) scores (OR = 1.050; 95% CI = 1.008–1.094; *p* value = 0.019) were independent risk factors for late seizures.

**Conclusion:**

Our study revealed that receiving MIS did not increase the incidence of late seizures after ICH. Additionally, cortical involvement and NIHSS scores were independent risk factors for late seizures in MIS-treated patients.

## Introduction

More than 5 million events of incident hemorrhagic stroke occur annually worldwide (1), and less than 50% of patients with intracerebral hemorrhage (ICH) survive to 1 year (2). Theoretically, surgical hematoma evacuation is intuitively appealing to reduce the mass effect and secondary cytotoxic injury of ICH (3, 4). However, positive effects may be offset by surgical trauma. Randomized controlled trials (RCTs) have shown that standard surgical procedures (open craniotomy) have not demonstrated improved functional outcomes or mortality in patients with ICH (5, 6). In recent years, surgical and radiologic technology have made minimally invasive surgery (MIS) possible (7). A meta-analysis has demonstrated that MIS for hematoma evacuation could decrease the rate of moderate to severe functional impairment and mortality in patients with supratentorial ICH (8). In addition, the results of minimally invasive catheter evacuation followed by thrombolysis (MISTIE) III trial showed that MISTIE group had a significant lower serious adverse events rate than standard medical care group, while the occurrence rates of symptomatic bleeding and brain bacterial infections were similar between two groups (9). Therefore, the bright prospect of MIS in ICH patients has renewed interest in ICH surgery.

Seizures are a common complication after intracerebral hemorrhage (ICH), affecting up to 6%–15% of patients with ICH in the acute and long-term periods (10, 11). Seizures after ICH can hurt the poststroke outcome and quality of life (12, 13); therefore, identifying individuals at high risk of seizures after a stroke and preventing epilepsy in a timely manner are crucial.

Previous studies have shown several risk factors for late seizures after ICH, such as cortical involvement, early seizures, a larger hemorrhage volume, a younger age of ICH onset, conventional surgical interventions, intraventricular hemorrhage, and the NIHSS score (10, 14–18). However, the association between minimally invasive hematoma aspiration and thrombolysis and late seizures after ICH remains uncertain. Therefore, in this study, we aimed to investigate whether MIS increases the risk of late seizures after ICH and use propensity score matching to eliminate interference from other risk factors. Additionally, a case–control study of MIS-treated patients with and without late seizures was performed to identify the risk factors for late seizures after ICH in this patient subgroup.

## Materials and methods

### Study design and subjects

This study was conducted at two tertiary hospitals in China–Tongji Hospital, Tongji Medical College, Huazhong University of Science and Technology (TJH) in Wuhan, and the First Affiliated Hospital of Wenzhou Medical University (WZH) in Wenzhou. Tertiary hospitals in China are medical centers with comprehensive medical, teaching and scientific research capabilities, and they provide medical and health services across regions, provinces, cities and across the country. We retrospectively enrolled consecutive inpatients diagnosed with ICH in the neurology department of TJH from January 2012 to December 2018 and of WZH from February 2014 to January 2020.

We did not include nonparenchymal hemorrhage or hemorrhagic transformation after cerebral infarction. The exclusion criteria were as follows: (1) patients with a history of multiple strokes; (2) patients with a history of epilepsy or other coexisting diseases contributing to epilepsy (e.g., brain tumor, traumatic brain injury, and intracranial vascular malformation); (3) patients without available computed tomography (CT) within 72 h after the onset of cerebrovascular symptoms; (4) patients who received craniotomy or other neurosurgery except minimally invasive hematoma aspiration and thrombolysis; (5) patients without late seizures who died within 1 year of ICH; (6) patients who were lost to follow-up (cannot be contacted during the period of the telephone interview) or refused to participate in the study.

According to the therapeutic intervention, the subjects were divided into the MIS group (ICH patients who received MIS including hematoma aspiration and thrombolysis) and conservative treatment group (ICH patients who received conservative medication). Because of the different therapeutic interventions and practice patterns for ICH during the trial period between the two hospitals, the MIS group was only derived from the patients of one hospital (TJH), while the conservative treatment group was derived from the patients of both hospitals (TJH and WZH). The procedure of hematoma aspiration and thrombolysis in this study has been presented in our previous publication (19). The study was approved by the Ethics Committee of Huazhong University of Science and Technology (ChiCTR- ROC- 2000039365) and Ethics Committee of the First Affiliated Hospital of Wenzhou Medical University (No. 2020–185). Verbal informed consent was obtained from the subject or subject's legally authorized representative by tele-interview, and the information of verbal consent was recorded in the consent forms.

### Clinical data on admission

In this study, a standardized data collection protocol was used to gather information on admission. Data items included demographics, lifestyle, medical history, clinical characteristics of ICH on admission, and imaging findings of ICH. The detailed data collection was as follows: (1) demographics, including age of ICH onset and sex; (2) lifestyle, including alcohol abuse and smoking status at stroke onset; (3) medical history, including hypertension, diabetes mellitus, hyperlipidemia, hyperuricemia and cardiac disorders (including atrial fibrillation and ischemic heart disease); (4) clinical characteristics of ICH on admission, including the National Institutes of Health Stroke Scale (NIHSS) score and Glasgow Coma Scale (GCS) score registered routinely by the treating physician on admission; (5) imaging findings of ICH, including the volume of ICH (measured using the ABC/2 method (20)), cortical involvement, multilobar involvement, and presence of intraventricular hemorrhage. All the images were read and reviewed by expert radiologists at two hospitals.

### Clinical follow-up and recognition of seizures

According to the definition for acute symptomatic seizures of the International League Against Epilepsy (ILAE), seizures after ICH were classified as early seizures (seizures that occurred within 7 days post-stroke) or late seizures (spontaneous unprovoked seizures that occurred beyond 7 days post-stroke) (21). Early seizures were extracted from inpatient medical records, while late seizures were identified by medical records and tele-interview based on a standardized questionnaire for screening late seizures (Supplementary Table S1) (22). The follow- up was conducted by a telephone interview and ended at death or in January 2020 (TJH) and October 2021 (WZH). The recognition of late seizures rested largely on patient and caregiver reports. Information obtained from the tele-interview was corroborated with past clinical records. Only events with clear focal motor or focal to bilateral tonic-clonic semiology by medical records or standardized questionnaire were included in the study. Given the retrospective nature of the study, potential nonmotor seizure events were conservatively excluded to reduce the risk of erroneously including ambiguous, non-seizure events. In addition, The MIS-treated patients received routine postoperative CT imaging during hospitalization, and the interval of CT imaging was 0 h, 12 h, 24 h, 48 h, 72 h, and 120 h after surgery.

### Propensity score matching

Propensity score matching was performed to balance the demographic and clinically relevant variables between the MIS and conservative treatment groups. The covariates for the propensity score matching were selected according to possible risk factors for ICH-related late seizures by previous studies (10). In this study, propensity scores for all the patients were estimated by multiple logistic regression models using the following covariates: age of ICH onset, history of alcohol abuse, NIHSS score, hemorrhage volume, cortical involvement, intraventricular hemorrhage, and presence of early seizures. The MIS and conservative treatment groups were matched 1:1 according to the nearest neighbor method (caliper was set at 0.03). Participants were only included in the comparison between groups when a suitable pairing was found.

### Statistical analysis

Before and after matching, between-group comparisons of the incidence of late seizures were performed between the MIS and conservative treatment groups. Univariate and multivariate stepwise backward logistic regression analyses were used to identify independent risk factors for late seizures in ICH patients taking MIS for hematoma evacuation. Only variables with *p* < 0.1 in univariate analysis were entered into the multivariate logistic regression model, and significant independent risk factors associated with late seizure after ICH were identified by backward elimination (*p* < 0.05). Categorical demographic and clinical variables were analyzed using chi-squared test or Fisher's exact test. The continuous demographic and clinical variables were analyzed by the Mann–Whitney U test. The level of statistical significance was set at *p* < 0.05 (two-tailed). All statistical analyses and propensity score matching were performed using Statistical Package for the Social Sciences version 26.0 (IBM Corporation, NY, USA).

## Results

**Figure 1:**
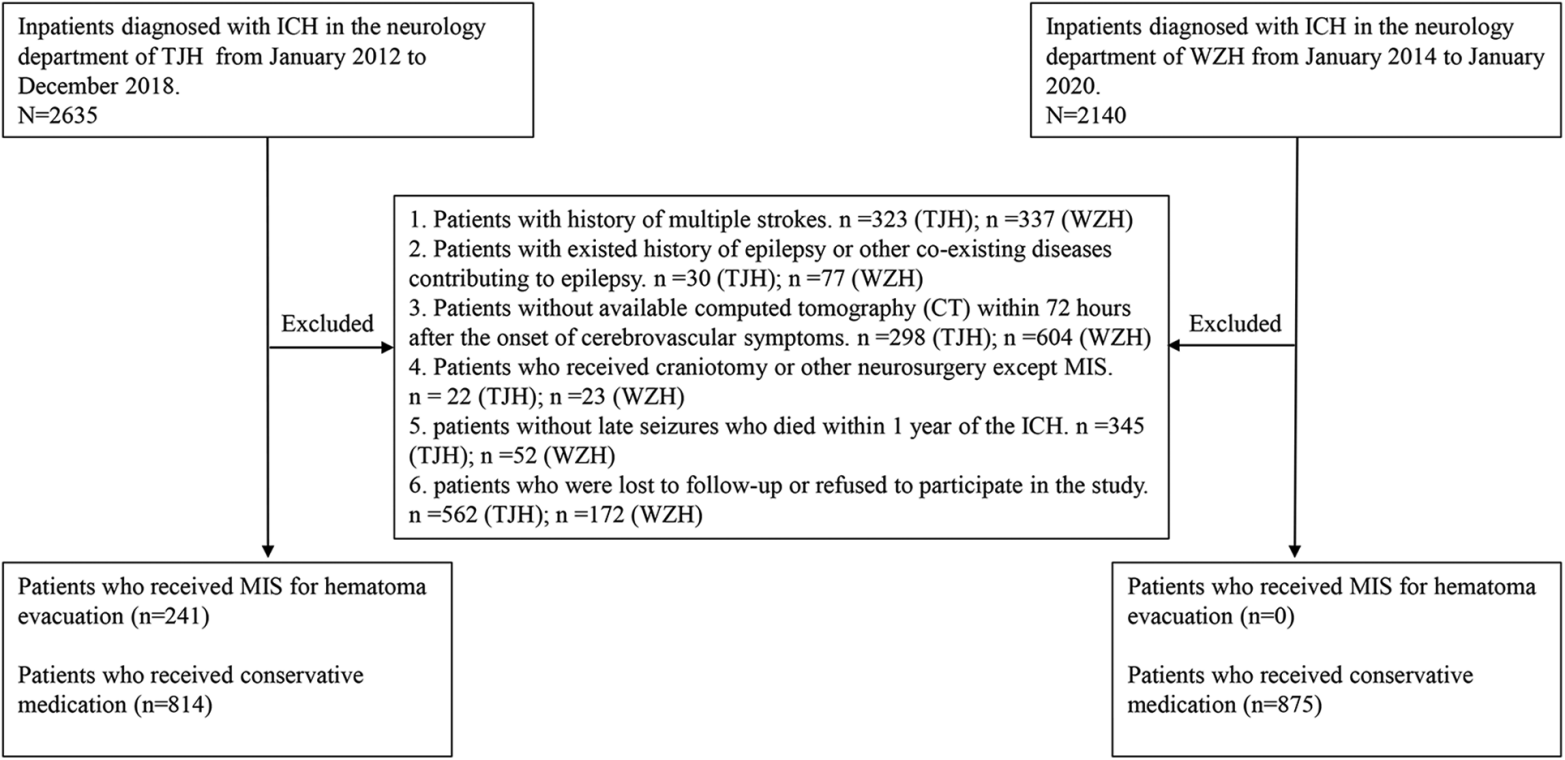
Flow chart of patient inclusion and exclusion. ICH = intracerebral hemorrhage; MIS = minimally invasive surgery; TJH = Tongji Hospital, Tongji Medical College, Huazhong University of Science and Technology; WZH = the First Affiliated Hospital of Wenzhou Medical University.

Figure 1 shows the flowchart of the selection of the subjects based on the inclusion/exclusion criteria. A total of 1,055 and 875 patients eligible for this study were derived from TJH and WZH, respectively. Additionally, 241 patients received MIS for hematoma evacuation (MIS group) and 1,689 patients chose conservative medication (conservative treatment group). The median follow-up time for all subjects was 48 months (interquartile range [IQR] = 31–68 months). The rates of late seizures were 12.9% (31/241) and 4.1% (69/1689) in the MIS group and conservative treatment group, respectively.

Propensity score matching was applied to balance the demographic and clinically relevant variables between the MIS and conservative treatment groups; 161 ICH patients from the MIS group were successfully matched with 161 ICH patients from the conservative treatment group (1:1). The demographic and clinically relevant variables before and after matching between the groups are shown in Table 1. Before matching, significant differences (*p* < 0.05) were found between the MIS and conservative treatment groups in the age of ICH onset, comorbidities (hypertension, diabetes mellitus, hyperlipidemia, hyperuricemia and cardiac disorders), NIHSS score, GCS score, volume of ICH, cortical involvement, multilobar involvement, intraventricular hemorrhage and follow-up duration. After matching, no significant differences (*p* > 0.05) were found in the covariates of propensity score matching (age of ICH onset, history of alcohol abuse, NIHSS score, volume of ICH, cortical involvement, intraventricular hemorrhage, and early seizures) or the remaining variables.

**Table 1:** Demographic and clinically relevant variables in patients with ICH before and after matching.

	Before matching			After matching		
	MIS group (*n* = 241)	Conservative treatment group (*n* = 1689)	*P* value	MIS group (*n* = 161)	Conservative treatment group (*n* = 161)	*P* value
Demographic variables						
Sex			0.874^a^			0.400^a^
Male	162 (67.2)	1,144 (67.7)		107 (66.5)	114 (70.8)	
Female	79 (32.8)	545 (32.3)		54 (33.5)	47 (29.2)	
Age of ICH onset (years)	50.0 (45.0–58.0)	57.0 (49.0–65.0)	<0.001^b^	50.0 (45.0–58.0)	52.0 (44.0–59.0)	0.807^b^
Lifestyle-related variables						
Alcohol abuse	76 (31.5)	567 (33.6)	0.531^a^	50 (31.1)	51 (31.7)	0.904^a^
Smoking	79 (32.8)	610 (36.1)	0.312^a^	49 (30.4)	57 (35.4)	0.343^a^
Comorbidities						
Hypertension	163 (67.6)	1,367 (80.9)	<0.001^a^	110 (68.3)	119 (73.9)	0.268^a^
Diabetes mellitus	12 (5.0)	250 (14.8)	<0.001^a^	11 (6.8)	18 (11.2)	0.173^a^
Hyperlipidemia	7 (2.9)	233 (13.8)	<0.001^a^	5 (3.1)	8 (5.0)	0.396^a^
Hyperuricemia	31 (12.9)	145 (8.6)	0.031^a^	19 (11.8)	20 (12.4)	0.864^a^
Cardiac disorders	10 (4.1)	199 (11.8)	<0.001^a^	8 (5.0)	10 (6.2)	0.628^a^
Clinical variables						
NIHSS score at admission	16.0 (12.0–24.0)	6.0 (2.0–11.0)	<0.001^b^	14.0 (11.0–19.5)	15.0 (9.5–22.0)	0.895^b^
GCS score at admission	12.0 (9.0–14.0)	15.0 (14.0–15.0)	<0.001^b^	13.0 (10.0–14.0)	13.0 (10.0–15.0)	0.685^b^
Early seizures	8 (3.3)	47 (2.8)	0.639^a^	8 (5.0)	6 (3.7)	0.585^a^
Late seizures	31 (12.9)	69 (4.1)	<0.001^a^	17 (10.6)	16 (9.9)	0.854^a^
Radiological variables						
Volume of ICH (ml)	35.3 (25.1–48.6)	8.4 (3.6–15.9)	<0.001^b^	29.6 (22.1–39.8)	28.3 (18.5–40.4)	0.188^b^
Cortical involvement	102 (42.3)	518 (30.7)	<0.001^a^	67 (41.6)	66 (41.0)	0.910^a^
Multilobar involvement	60 (24.9)	194 (11.5)	<0.001^a^	43 (26.7)	41 (25.5)	0.800^a^
Intraventricular hemorrhage	83 (34.4)	378 (22.4)	<0.001^a^	47 (29.2)	40 (24.8)	0.380^a^
Follow-up duration (months)	44.9 (28.9–63.0)	48.2 (31.2–69.3)	0.008^b^	41.4 (28.1–57.0)	46.4 (27.6–66.6)	0.093^b^

ICH, intracerebral hemorrhage; MIS, minimally invasive surgery; NIHSS, national institutes of health stroke scale; GCS, glasgow coma scale. All values are expressed as numbers (%) or medians (interquartile range).

^a^ Chi-squared test.

^b^ Mann–Whitney *U* test.

**Figure 2:**
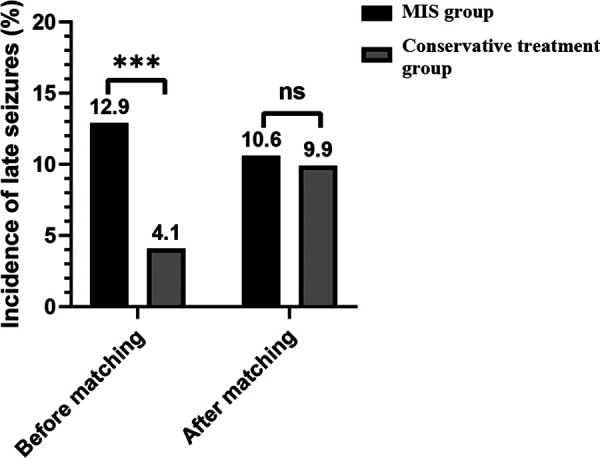
Comparison of the late seizure rates between the MIS and conservative treatment groups. MIS = minimally invasive surgery; *** = *p* value < 0.001; ns = nonsignificance.

Figure 2 compares the late seizure rates between the MIS and conservative treatment groups. Before matching, significant differences (*p* < 0.001) were found between the MIS group (31/241; 12.9%) and conservative treatment group (69/1689; 4.1%) in the incidence of late seizures. However, after matching, no significant differences (*p* = 0.854) were found between the MIS group (17/161; 10.6%) and conservative treatment group (16/161; 9.9%).

Among the participants in TJH, 96 patients (9.1%) received regular blood thinning/antiplatelet drugs during the follow-up. The rates of late seizures were 3.1% (3/96) in the patients receiving blood thinning/antiplatelet drugs, while 5.5% (53/959) in the patients without taking those drugs, and no significant differences (*p* = 0.317) were found between the two groups. In the MIS group, 31 patients (12.9%) had one or more seizures beyond 7 days post-ICH. Table 2 provides the baseline characteristics of the MIS group with and without late seizures. Univariate analysis showed that cortical involvement, NIHSS scores at admission and the volume of ICH were associated with late seizures (*p* < 0.1). Multivariate stepwise logistic regression analysis revealed that only cortical involvement (OR = 2.547; 95% CI = 1.137–5.705; *p* value = 0.023) and higher NIHSS scores at admission (OR = 1.050; 95% CI = 1.008–1.094; *p* value = 0.019) were independent risk factors for the occurrence of late seizures.

**Table 2:** Univariate and multivariate analysis of factors associated with late seizures in ICH patients who received MIS for hematoma evacuation.

	Univariate analysis			Multivariate analysis	
	Patients with late seizures (*n* = 31)	Patients without late seizures (*n* = 210)	*p* value	OR (95% CI)	*p* value
Demographic variables					
Sex			0.245^a^		
Male	18 (58.1)	144 (68.6)			
Female	13 (41.9)	66 (31.4)			
Age (years)	49.0 (45.0–57.0)	50.0 (45.0–58.3)	0.358^c^		
Lifestyle-related variables					
Alcohol abuse	11 (35.5)	65 (31.0)	0.612^a^		
Smoking	9 (29.0)	70 (33.3)	0.634^a^		
Comorbidities					
Hypertension	20 (64.5)	143 (68.1)	0.691^a^		
Diabetes mellitus	0 (0)	12 (5.7)	0.373^b^		
Hyperlipidemia	0 (0)	7 (3.3)	0.600^b^		
Hyperuricemia	5 (16.1)	26 (12.4)	0.567^b^		
Cardiac disorders	2 (6.5)	8 (3.8)	0.622^b^		
Clinical variables					
NIHSS score at admission	20.0 (12.0–35.0)	15.0 (12.0–22.0)	0.027^c^	1.050 (1.008–1.094)	0.019
GCS score at admission	10.0 (8.0–14.0)	12.0 (9.8–14.0)	0.119^c^		
Early seizures	2 (6.5)	6 (2.9)	0.274^a^		
Radiological variables					
Volume of ICH (ml)	40.2 (30.1–54.4)	34.6 (24.4–47.8)	0.046^c^	1.010 (0.992–1.028)	0.292
Cortical involvement	19 (61.3)	83 (39.5)	0.022^a^	2.547 (1.137–5.705)	0.023
Multilobar involvement	11 (35.5)	49 (23.3)	0.144^a^		
Intraventricular hemorrhage	10 (32.3)	73 (34.8)	0.784^a^		
Postoperative rebleeding	5 (16.1)	21 (10.0)	0.305^a^		

ICH, intracerebral hemorrhage; MIS, minimally invasive surgery; OR, odds ratio; 95% CI, 95% confidence interval; NIHSS, national institutes of health stroke scale; GCS, glasgow coma scale. All values are expressed as numbers (%) or medians (interquartile range).

^a^ Chi-square test.

^b^ Fisher's exact test.

^c^ Mann–Whitney *U* test.

## Discussion

In the present study, the incidence of late seizures in ICH patients who received MIS was significantly higher than that in ICH patients who received conservative medication (12.9% vs. 4.1%; *p* value < 0.001). However, substantial differences were found between the two patient subgroups, including those with proven risk factors for late seizures after ICH. To eliminate these interferences, propensity score matching was performed to balance relevant risk factors between the MIS and conservative treatment groups. After matching, no significant differences were found in those with proven risk factors between the subgroups (Table 1), and no significant differences were found between the patients with MIS and those with conservative medication in the incidence of late seizures in the ICH patients (10.6% vs. 9.9%; *p* value = 0.854). Additionally, our study showed that a higher NIHSS score at admission and cortical involvement were independent risk factors for late seizures in ICH patients with MIS.

In recent years, several trials have shown that craniotomy does not improve the functional outcome or mortality of ICH, likely because the surgical approach creates sufficient trauma to the surrounding brain for hematoma evacuation (5, 6). Previous studies have explored the relationship between conventional surgical hematoma evacuation and late seizures (15, 17). A prospective study of spontaneous intracerebral hemorrhage found that conventional surgical hematoma evacuation was an independent risk factor for late seizures (OR = 2.6; 95% CI = 1.4–4.8; *p* value = 0.0027) (15). Another study showed that hematoma evacuation, which included standard craniotomy and MIS, increased the risk of late seizure after ICH (OR = 2.47; 95% CI = 1.12–5.45; *p* value < 0.05) (17). Additionally, other frequent complications reported in ICH patients with craniotomy were pulmonary (30.4%), renal (3.2%), and thromboembolic (2.9%) complications (23).

The failure of conventional surgical interventions and common complications leaves MIS as the most promising surgical strategy for ICH patients. MIS for ICH aims to reduce the manipulation of healthy brain tissue, decrease the procedure time and achieve rapid hematoma evacuation (24). A meta-analysis demonstrated that MIS is associated with lower rates of moderate-to-severe functional impairment and lower mortality than medical management or conventional surgery (25). Nevertheless, previous studies were mostly small, unblinded, and none assessed the association between surgical performance and clinical outcome. So additional evidence is needed to recommend use of minimally invasive surgery plus thrombolysis in routine care. MISTIE III was an open-label, blinded endpoint, phase 3 trial and the trial centrally randomized patients to MISTIE treatment group or standard medical care group (9). The result of MISTIE III trial showed that MISTIE did not improve functional outcomes compared with standard medical care in patients with large intracerebral hemorrhage. However, assessment of the surgical data indicates that when MISTIE achieves the protocol-defined surgical aim, which include large reduction of hematoma size to 15 ml or less, a benefit in mortality and functional improvement seems possible. More importantly, the MISTIE procedure was safe with regard to serious bleeding and infection, and MISTIE group had a significant lower serious adverse events rate than standard medical care group. These findings suggest MISTIE has few negative consequences. Our study showed that receiving MISTIE did not increase the incidence of late seizures after ICH, providing another evidence for safety of MISTIE.

The reported incidence of late seizures after ICH ranges from 3.1% to 10.8% (14–18, 26, 27). In this study, the incidence of late seizures in ICH patients with MIS was up to 12.9% (31/241); in patients with conservative medication, it was 4.1% (69/1689). The main reason for the high incidence of late seizures was that MIS-treated patients had more risk factors for late seizures (10, 14, 16), including significantly younger ages, higher NIHSS scores, a larger volume of ICH, and more frequent cortical involvement and intraventricular hemorrhage than those treated conservatively (Table 1). After eliminating interferences by propensity score matching, MIS for hematoma evacuation showed no significantly higher risk of late seizures than conservative medication (Figure 2). This result may be attributed to the reduction in excitotoxic glutamate concentrations and improvement in blood brain barrier integrity within the perihematoma region by MIS (28, 29).

In our analysis of the patients with MIS, the risk of developing late seizures after ICH is increased with cortical involvement and higher NIHSS scores. Cortical involvement has been consistently reported as a risk factor for post-ICH seizures (14, 15, 17, 26). Gliosis, neuronal loss, hemosiderin deposition, neurodegeneration and altered synaptic plasticity in neurons near the cortical area lead to greater hyperexcitability of synaptic activities than that in the deep part of the brain parenchyma (30). The association between cortical involvement and seizures may be attributed to the increase in neuronal excitability and disinhibition by altering the cortical structure and cortical compression (31). Likewise, several previous studies have already established higher NIHSS scores as a risk factor for late seizure development (16, 22, 32). In the current era of personalized medicine, the diagnosis or prognosis of disease has become increasingly significant (33). The CAVE score was the first tool to estimate the risk of late seizures after ICH in individual patients, and the score was derived using a 0–4 point scoring system with 1 point each for cortical involvement, age <65 years, volume >10 ml, and early seizures within 7 days of ICH (14). The c-statistic of the CAVE score was 0.81 (0.76–0.86) and 0.69 (0.59–0.78) in the internal and external validation cohorts, respectively. Among the clinical scores currently available, the CAVS score was the only score that included surgical variables, improving the CAVE score by substituting surgical evacuation for early seizures (15). Our study showed that MIS did not significantly increase the risk of late seizures; therefore, MIS should be separated from conventional surgery in terms of clinical scores instead of being a risk factor.

Our study has several limitations. First, as our study design was retrospective, identifying patients with early seizures relied on inpatient medical records. Although motor seizures could be correctly identified by the treating clinician and the researchers, there was less certainty around the recognition for patients with subtle, non-motor seizures. The latter events were excluded from the study, therefore, it inevitably led to the under-reporting early seizure events. In addition, registering seizure events retrospectively is prone to misdiagnosis and underdiagnosis, although the medical reports were collected based on a standardized questionnaire. Second, as the recognition of late seizures rested largely on patient and caregiver reports in the tele-interview, we did not have data on subclinical electrographic seizures. Therefore, the incidence of late seizures may have been underestimated. Given the high early mortality associated with ICH, competing risk from death not related to the outcome of interest is a potential problem in this study and represents an inevitable bias. Furthermore, the conservative treatment group consists of the patients who were not deemed eligible for surgery, so the potential bias of the propensity score matching is inevitable due to the absence of choosing patients randomly for surgery.Despite these limitations, our study provided an independent cohort including MIS-treated patients, as well as a large two-center control cohort including medication-treated patients for strict matching. To our knowledge, this study is the first on the association between MIS and late seizures after ICH.

## Conclusion

In summary, our study revealed that receiving MIS did not increase the incidence of late seizures after ICH. Additionally, cortical involvement and NIHSS scores were independent risk factors for late seizures in MIS-treated patients.

## Data Availability

The raw data supporting the conclusions of this article will be made available by the authors, without undue reservation.
