# Supplementary material for: Association between minimally invasive surgery and late seizures in patients with intracerebral hemorrhage: A propensity score matching study
Source: Front Surg. 2022 Oct 13;9:949804. doi: 10.3389/fsurg.2022.949804 (PMC9606625; doi:10.3389/fsurg.2022.949804)
Supplement: Supplementary file 1 [file Table1.docx]

Table S1. Late seizures screening questionnaire.

| Q1: Self-reported diagnosis  a. Has ever been diagnosed by a doctor to have seizures/epilepsies/convulsions after discharge? ^a^  b. How soon after discharge did you experience the first recurrent seizure? ^b^  Only patients responded Q1a with “yes” were recorded as having late seizures, if not, patients were asked by following questions. |
| --- |
| Q2: Symptom-based screening questions: a, b  a. Have you ever had, or has anyone told you that you had any of the following symptom after discharge? ^c^  i. A seizure, convulsive, fit or spell under any circumstances?  ii. Uncontrolled movement of part or all of your body such as twitching, jerking, shaking, or going limp?  iii. An unexplained change in your mental state or level or unawareness; or an episode of “spacing out” that you could not control?  iv. Shortly after waking up, either in the morning or after a nap, have you ever noticed uncontrollable jerking or clumsiness, such as dropping things or things suddenly “flying” from your hands?  v. Have you ever had repeated unusual spells?  b. How soon after discharge did the symptom happen? ^b^ |
| Q3: Questions about Anti-seizure medications?  a: Are you currently using any anti-seizure drug including “valproate,” “levetiracetam,” “carbamazepine,” or “oxcarbazepine”?  b: If not, when did you stop the drug? |

a Acceptable answers to each of the questions include: “yes,” “no,” “possible,” or “don’t know.”

b Only if the patient or caregiver can provide the approximate date or we can obtain it from the medical records, he/she were included in analysis.

c Patients were diagnosed with late seizures only if they fulfill 1 + 2 + 3 or 1 + 4 of the following: 1. One or more “yes” for Q2a-i to v, 2. Provide an approximate date for Q2b, 3. “yes” for Q3a, 4. confirmed by medical records.
